# Does Time Matter in Deficit of Calcium after Total Thyroidectomy in Subjects with Previous Bariatric Surgery?

**DOI:** 10.3390/nu14091805

**Published:** 2022-04-26

**Authors:** Salvatore Tramontano, Gerardo Sarno, Pietro Calabrese, Luigi Schiavo, Maria Spagnuolo, Vincenzo Pilone

**Affiliations:** 1 Center of Excellence of Bariatric Surgery of the Italian Society of Obesity Surgery and Metabolic Disease (SICOB), Unit of General and Emergency Surgery, University Hospital San Giovanni di Dio e Ruggid’Aragona, P.O. Gaetano FucitoMercato San Severino, 84085 Salerno, Italy; salvytra@libero.it (S.T.); pietrocalabres@gmail.com (P.C.); lschiavo@unisa.it (L.S.); mariaspagnuolo94@gmail.com (M.S.); vpilone@unisa.it (V.P.); 2 General Surgery and Kidney Transplantation Unit, “San Giovanni di Dio e Ruggi D’Aragona” University Hospital, Scuola Medica Salernitana, 84125 Salerno, Italy; 3 Department of Medicine, Surgery and Dentistry, “Scuola Medica Salernitana”, University of Salerno, Baronissi, 84081 Salerno, Italy

**Keywords:** chrononutrition, bariatric surgery, thyroidectomy, hypocalcemia, hypoparathyroidism, obesity

## Abstract

Background: Hypoparathyroidism-related hypocalcemia is a common complication after total thyroidectomy (TT), particularly if there is a history of prior bariatric surgery. However, it is still unknown if it is the surgery timing or the type of bariatric intervention that increases the risk of developing this complication. Methods: We compared the risk of hypocalcemia (serum calcium levels < 8 mg/dL) and hypoparathyroidism (both transient and permanent) between patients with restrictive procedures (LSG and GB) and patients without a history of obesity surgery in the immediate post-operative period and after 12 months. Hypoparathyroidism was considered permanent if the plasma parathyroid hormone (PTH) levels at 6 months were less than 15 pg/mL and the patient still required oral calcium (calcium carbonate) and vitamin D supplementation, in addition to the supplements that were taken routinely before thyroidectomy. Results: From the 96 patients who underwent TT, 50% had a history of bariatric surgery: 36 LSG and 12 GB. The risk of hypocalcemia was similar in patients with a history of restrictive procedures (31.35%) and in controls (25%) (*p* = 0.49). Furthermore, hypocalcemia risk was similar between patients with a history of LSG (30.5%) and GB (33%) (*p* = 0.85). The prevalences of transient and permanent hypoparathyroidism were similar between patients with a history of restrictive procedures and in controls; similarly, no differences were detected between subjects undergoing LSG and GB. Conclusions: Restrictive bariatric surgery (LSG and GB) is not a risk factor for post-thyroidectomy hypocalcemia and hypoparathyroidism and thus did not require a different perioperative supplementation protocol compared to subjects without history of bariatric surgery undergoing TT.

## 1. Introduction

Hypocalcemia is the most common complication after thyroidectomy, with variable incidence in literature (3–20%), associated with transient or definitive hypoparathyroidism (hypo-PTH) [1]. Severe and definitive hypo-PTH ranges between 1–3% [2,3]. Knowledge of all clinical and biochemical risk factors is useful for providing correct information to patients and for supporting preventive measures, and it is also associated with determining appropriate surgical techniques [4,5]. Female sex, autoimmune thyroid disease, hyperthyroidism, low pre-operative vitamin D level, bilateral central neck dissection, or inadvertent excision of parathyroid glands are all risk factors for post-operative hypocalcemia [5,6,7]. The prevalence of severe and morbid obesity (corresponding to a body mass index (BMI) > 35kg/m^2^ and 40 kg/m^2^, respectively) has increased as well [5,8]. Bariatric surgery, meanwhile, has emerged as the only effective and long-term treatment of morbid obesity, with subsequent improvement or remission of comorbid diseases [9,10,11]. Recently, a few studies have reported bariatric surgery as a risk factor for hypocalcemia in patients who underwent thyroidectomy [12,13]. Indeed, a study was carried out in 48 subjects who had previously undergone obesity surgery (i.e., Roux-en-Y gastric bypass (RYGBP), sleeve gastrectomy, or adjustable gastric band) and subsequently underwent total thyroidectomy, aiming to evaluate incidence and factors involved in the risk of hypocalcemia (transient and permanent) and the post-operative outcomes of these patients after total thyroidectomy [12]. The main finding of this study was that especially after Roux-en-Y gastric bypass, patients have an increased risk for hypocalcemia after total thyroidectomy [12]. Another study was carried out in 14 patients who underwent total thyroidectomy with a history of preceding RYGBP that were matched 2:1 for age, gender, and BMI to a control group (*n* = 23) undergoing total thyroidectomy without previous RYGBP during the same study period [13]. A comparison between groups demonstrated that patients who underwent total thyroidectomy with a history of preceding RYGBP had a significantly higher incidence of symptomatic hypocalcemia resulting in paresthesia and tetany, and that these patients received more intravenous calcium and had longer hospital stays than controls [13]. The above reported evidence mostly concerns subjects undergoing malabsorptive surgery; however, no evidence has been reported regarding subjects undergoing restrictive bariatric procedures. Thus, the aims of our study were as follows: (1) to compare the prevalence of post-operative hypocalcemia associated with hypo-PTH (transient and permanent) after total thyroidectomy (TT) in patients with previous history of restrictive bariatric surgery (laparoscopic sleeve gastrectomy (LSG) and gastric binding (GB)) compared with a cohort of patients without; and (2) to compare the prevalence of post-operative hypocalcemia associated with hypo-PTH (transient and permanent) after TT in patients undergoing LSG compared to subjects undergoing to GB.

## 2. Methods

### 2.1. Design and Setting

This observational and cross-sectional retrospective study was carried out in patients referred for surgical treatment of thyroid diseases from 1 January 2017 to 31 August 2017 at the Center of Excellence of Bariatric Surgery of the Italian Society of Obesity Surgery and Metabolic Disease (SICOB), Unit of General and Emergency Surgery, University Hospital San Giovanni di Dio e Ruggi d’Aragona, Mercato San Severino, Salerno. The study was carried out in accordance with the Code of Ethics of the World Medical Association (Declaration of Helsinki) for experiments that involve humans. Due to its retrospective nature, ethics approval was not necessary. The aim of the study was clearly explained to all study participants. Each subject gave her/his informed consent to the collection of data, and the review board of our hospital gave consent to revise the collected data. According to The National Code on Clinical Trials, ethics approval is not necessary for retrospective studies [14] (see the National Code on Clinical Researches published in Official Gazette numbered 28030 from 13 April 2013). Written informed consent was obtained. Data were collected anonymously and recorded in a prospectively maintained database. In order to assess the presence of permanent hypo-PTH, patients were asked if they were taking oral calcium and/or vitamin D supplements to maintain serum calcium in the normal range.

### 2.2. Study Population

Ninety-six patients (>18 years of age) referred to our surgical outpatient clinic for surgical treatment of thyroid diseases were enrolled after providing written informed consent. Forty-eight of them previously underwent restrictive bariatric surgery at least 12 months before TT (Group 1), whereas 48 did not and were considered as controls (Group 2). 

Inclusion criteria were as follows: (1) thyroid diseases candidate for thyroidectomy, such as multinodular or nodular goiter with compressive symptoms; (2) cancerous multinodular or nodular goiter according to the Italian cytological classification [15]. Eligible participants for the study were adult subjects aged 18–75 years with normal liver, cardiopulmonary, and kidney functions, as determined by medical history, physical examination, electrocardiogram, urinalysis blood tests for blood urea nitrogen, creatinine, uric acid, albumin, aspartate aminotransferase, and alanine aminotransferase. Other exclusion criteria were chronic diseases that could interfere with calcium homeostasis, altered levels of serum calcium or albumin, current therapy with osteoporosis therapies, and medications that may affect vitamin D absorption or metabolism, including anti-inflammatory drugs, sex hormone therapy, statins and other hypolipidemic agents, medications to reduce body weight, pregnancy, breastfeeding, subtotal or unilateral thyroidectomy, history of neck irradiation, hyperthyroidism, and previous surgery for hyperparathyroidism. 

### 2.3. Anthropometric Measurements 

Subjects dressed in light clothes and no shoes underwent the assessment of anthropometric parameters. The formula for the calculation of BMI was as follows: weight (kg)/height (m^2^). To evaluate height, a wall-mounted stadiometer was used, while body weight was assessed using a calibrated scale.

### 2.4. Laboratory Test

After fasting overnight, venous blood samples were collected from the antecubital vein and were stored in vacutainer tubes containing ethylenediaminetetraacetic acid (EDTA). Samples were collected in the morning between 8:00 a.m. and 12:00 p.m. after an overnight fast of at least 8 h. The samples were stored at −80 °C until processed. A direct competitive chemiluminescence immunoassay (CLIA) (Liaison, DiaSorin, Saluggia, Italy) was used to evaluate 25OHD levels, with a specificity of 100% for 25OHD. All biochemical parameters were assessed using a Roche Modular Analytics System. PTH levels were measured using an enzyme chemiluminescence immunoassay (Roche Products, Modular E, Penzberg, Germany). The laboratory tests were performed at the baseline. Serum calcium was assessed at the baseline, on post-operative day (POD) 1, and 12 months after surgery, and it was normed for albumin. Post-operative hypocalcemia was defined for values of SCC lower than 8 mg/dL on POD 1 (normal range, 8.4–10.5 mg/dL). PTH was measured at the baseline, on POD 2, and 12 months after surgery.

We decided to follow the patients up until 12 months because this interval was considered effective for stabilization of permanent hypo-PTH, and for recovery of transient hypo-PTH. 

Hypo-PTH was defined as permanent if the patient presented plasma PTH levels of 15 pg/mL for at least 12 months, requiring oral calcium and/or vitamin D supplements to maintain the serum calcium in the normal range.

### 2.5. Thyroidectomy

Pre-operative work-up was carried out at the surgical department. Indications for surgery were evaluated and approved by a multidisciplinary team consisting of surgeons, an anesthesiologist, an otolaryngologist, and a nutritionist who discussed each case. Other than blood samples, chest X-ray, and ECG, an indirect laryngoscopy in pre-operative phase was carried out in order to assess the local status of larynges and vocal cords. Total thyroidectomy was performed by experienced surgeons. The same team operated on all the patients. When needed, the procedure was completed with microsurgery assistance for correct visualization of recurrent nerve and parathyroid. as Additionally, for intervention, post-operative management of patients was carried out by experienced surgeons for bariatric and neck surgery, who followed standardized protocols, unchanged during the study period. Palsy of recurrent laryngeal nerve lasting since the second post-operative week was considered an indication for rehabilitation therapy, according to otolaryngology indications. Rehabilitation was continued up to relief of symptoms. 

### 2.6. Statistical Analysis

Continuous data are presented as mean, standard deviation (*SD*), and range. Qualitative data are represented as percentage or frequency of matched cohorts. Data were analyzed as appropriate by using the chi square (χ2) test for categorical variables, Student’s paired *t*-test for continuous variables, and the McNemar method for matched pairs. For this study, *p* < 0.05 was considered to be statistically significant. Statistical analysis was carried out by using SPSS^®^ version 13 (SPSS, Chicago, IL, USA).

In the absence of similar clinical studies available in the literature, the calculation of the sample size was performed a priori by considering the effect size 0.8 with type I error of 0.05 and a power of 90%. The number of subjects to be enrolled was found to be at least 34 per group. Since we enrolled more subjects than the necessary number, we decided to include all of them in the statistical analysis in order to strengthen the power of the results. The calculation of the sample size was performed using G Power Software.

## 3. Results

Among 96 patients who received TT during the study period, 48 patients presented with a history of bariatric surgery (Group 1) and 48 subjects did not (Group 2). In all cases, the procedure was performed with a standardized technique, using mini-cervicotomy. All patients completed a 12-month follow-up. 

There were no differences in the cases of thyroid surgery for malignancy (three cases in group 1 and two cases in group 2) between both groups. The two groups presented with no differences in the pre-operative clinical and biochemical characteristics (specifically the serum calcium and vitamin D levels). Patients with a history of restrictive bariatric surgery had a similar risk of post-operative hypocalcemia compared to subjects without previous history of bariatric surgery (31.25% vs. 25%; *p* = 0.49). Although there was a trend of a higher prevalence of transient and permanent hypoparathyroidism in group 1 compared to group 2, it did not reach statistical significance (Table 1). 

Upon comparing subjects undergoing LGB with subjects undergoing to GB, we found no significant differences in the rates of hypocalcemia, regarding both transient and permanent hypoparathyroidism (Table 2). 

However, we detected a significantly higher BMI value in subjects undergoing LSG compared to subjects undergoing GB (*p* = 0.0006).

## 4. Discussion

Our study confirmed that patients with a history of restrictive bariatric surgery have no increased risk for hypocalcemia and hypo-PTH after TT. This risk was recently evidenced in literature—both with comparative trials, and with case reports of recalcitrant hypocalcemia requiring reversal of bariatric procedure [12,13]. However, the majority of reports concerned patients with previous RYGB: this procedure is associated with variable rates of malabsorption, which requires calcium and vitamin D supplementation. In some cases, this therapy is stopped by patients without medical evaluation; in other cases, ineffective supplementation has been reported. Our study first specifically evidenced that patients previously submitted to restrictive bariatric surgery (GB and LSG) were no exposed to higher risk of post-operative hypocalcemia and hypo-PTH than matched patients without a history of bariatric surgery. As confirmed by a recent review on bariatric patients treated with TT, all patients with a history of previous bariatric surgery should be closely monitored, with serum calcium and PTH levels monitored for a longer period time, and a careful and prolonged supplementation should also be suggested in asymptomatic cases [16]. However, this is not the case of restrictive bariatric surgery; indeed, a metanalysis carried out in 120 patients did not confirm this risk in patients with previous GB and LSG, although the number of cases as well as the heterogeneity of studies is questionable [17]. This is due to the fact that these two procedures do not lead to malabsorption and do not damage the anatomy of the duodenum and the proximal jejunum, which have the highest concentration of calcium transporters, and thus, the main absorption sites of calcium [18]. In addition, restrictive bariatric surgery is based on restriction and hormonal changes that may influence hunger and satiety, and not malabsorption, thereby causing fewer nutritional or vitamin deficiencies [19]. 

Vitamin D deficiency is particularly common in subjects with obesity (50%) [20,21,22]. It depends on different factors, such as decreased expression of vitamin D metabolizing enzymes, decreased sun exposure, altered dietary habits, and lack of adequate amounts of various minerals related with vitamin D [22,23]. In fact, the reduced uptake of daily calories after bariatric surgery leads to a reduction of daily vitamin D. Furthermore, most bariatric patients also develop an intolerance to calcium-rich foods after surgery [24]. However, our enrolled subjects had normal levels of pre-operative vitamin D, which could be considered to be an additional protective factor against post-operative hypocalcemia. This is because according to the new guidelines of the American Society for Metabolic and Bariatric Surgery (ASMBS), after bariatric surgery, our patients took early and specific calcium and vitamin D supplementation (1.5 mg/d of oral calcium (in diet and as citrated supplement in divided doses) plus 3000 international units (IU) of vitamin D) [25].

There is no consensus on the prevention and management of hypocalcemia after TT in patients with a history of bariatric surgery [26]. Only a few case reports have emphasized the need for finding specific perioperative management approaches [27]. There has been only one study of 19 thyroid resections after RYGB, in which McKenzie reported a 42% risk of symptomatic hypocalcemia [28]. In another study, Durr recommended early prophylactic intravenous calcium administration within 1 h after the completion of TT for all patients with previous RYGB surgery and a different regimen of high-dose oral calcium and calcitriol after discharge, but these results did not demonstrate a standardization of approach [29]. However, our findings show that patients undergoing restrictive procedures such as LSG and GB carry the same risk for post-thyroidectomy hypocalcemia and hypoparathyroidism as patients without a history of bariatric surgery.

There are some limitations to our study due to its observational and retrospective nature that precludes causality-related information. However, this cohort of patients represents a homogeneous group who underwent standardized surgical procedures that were carried out by the very same operators at the very same center, thus minimizing any operator-related variability. 

## 5. Conclusions

Restrictive bariatric surgery is not a risk factor for post-operative hypocalcemia and hypoparathyroidism (both transient and permanent). Further evaluations in prospective studies and big data gathering are needed.

## Figures and Tables

**Table 1:** Anthropometric, perioperative, biochemical, and pathologic features of subjects with previous history of restrictive bariatric surgery (LSG + GB) (Group 1) and without (Group 2).

Parameters	Group 1 (*n* = 48)	Group 2 (*n* = 48)	*p*
Age (years)	39.77 ± 12.05	43 ± 11.18	0.18
M/F	12/36	14/34	X^2^ = 0.211 *p* = 0.64
Serum calcium (baseline) mg/dL	8.46 ± 0.34	8.53 ± 0.37	0.34
Serum calcium (POD 1) mg/dL	8.03 ± 0.59	8.19 ± 0.54	0.18
Post-operative hypocalcemia (%)	31.25%	25%	X^2^ = 0.4638 *p* = 0.49
Serum calcium (12 months)	9.01 ± 0.55	9.03 ± 0.57	0.91
25-OH-D (baseline) (ng/mL)	43.17 ± 12.02	44.32 ± 13.0	0.65
PTH (baseline)	44.86 ± 8.95	46.58 ± 9.72	0.40
PTH (POD 2)	14.02 ± 3.08	14.44 ± 2.96	0.95
PTH (12 months)	46.80 ± 16.88	56.94 ± 13.38	0.089
Patients with transient hypo-PTH (%)	14.5%	10.4%	X^2^ = 0.381 *p* = 0.53
Patients with permanent hypo-PTH (%)	4.1%	0%	X^2^ = 0.365 *p* = 0.54

M: males. F: Females. POD: post-operative day. PTH: parathormone.

**Table 2:** Comparison between LGB and GB patients according to anthropometric, peri-operative, biochemical, and pathologic features.

Parameters	LSG (*n* = 36)	GB (*n* = 12)	*p* Value
Age (years)	40.0 ± 12.27	39 ± 11.33	0.80
M/F	10/26	2/10	X^2^ = 0.5926 *p* = 0.44
BMI (kg/m^2^)	32.25 ± 3.23	35.85 ± 1.60	0.0006
Serum calcium (baseline) mg/dL	8.43 ± 0.32	8.54 ± 0.35	0.349
Serum calcium (POD 1) mg/dL	8.0 ± 0.61	8.12 ± 0.50	0.56
Post-operative hypocalcemia (%)	30.5%	33%	X^2^ = 0.032 *p* = 0.85
Serum calcium (12 months)	8.85 ± 0.46	9.45 ± 0.55	0.07
25-OH-D (baseline) (ng/mL)	44.9 ± 12.96	37.75 ± 5.93	0.07
PTH (baseline)	45.17 ± 8.11	44.35 ± 10.74	0.785
PTH (POD 2)	15.05 ± 3.2	13.05 ± 2.0	0.299
PTH (12 months)	42.98 ± 17.99	57.3 ± 5.34	0.168
Patients with transient hypo-PTH (%)	14%	25%	X^2^ = 0.8 *p* = 0.37
Patients with permanent hypo-PTH (%)	5.5%	0%	X^2^ = 0.0759 *p* = 0.78

LSG: laparoscopic sleeve gastrectomy. GB gastric bending.

## Data Availability

The data that support the findings of this study are available from the corresponding author, [G.S.], upon reasonable request.

## References

[1] Orloff L.A., Wiseman S.M., Bernet V.J., Fahey T.J., Shaha A.R., Shindo M.L., Snyder S.K., Stack B.C., Sunwoo J.B., Wang M.B. (2018). American Thyroid Association Statement on Postoperative Hypoparathyroidism: Diagnosis, Prevention, and Management in Adults. Thyroid Off. J. Am. Thyroid Assoc..

[2] Dedivitis R.A., Aires F.T., Cernea C.R. (2017). Hypoparathyroidism after thyroidectomy: Prevention, assessment and management. Curr. Opin. Otolaryngol. Head Neck Surg..

[3] Ponce de Leon-Ballesteros G., Bonilla-Ramirez C., Hernandez-Calderon F.J., Pantoja-Millan J.P., Sierra-Salazar M., Velazquez-Fernandez D., Herrera M.F. (2020). Mid-Term and Long-Term Impact of Permanent Hypoparathyroidism After Total Thyroidectomy. World J. Surg..

[4] Del Rio P., Sommaruga L., Bezer L., Arcuri M.F., Cataldo S., Ceresini G., Sianesi M. (2010). Preoperative PTH as a marker of risk for post-thyroidectomy hypocalcemia. Minerva Endocrinol..

[5] Tredici P., Grosso E., Gibelli B., Massaro M.A., Arrigoni C., Tradati N. (2011). Identification of patients at high risk for hypocalcemia after total thyroidectomy. Acta Otorhinolaryngol. Ital. Organo Uff. Della Soc. Ital. Di Otorinolaringol. E Chir. Cervico-Facciale.

[6] Antakia R., Edafe O., Uttley L., Balasubramanian S.P. (2015). Effectiveness of preventative and other surgical measures on hypocalcemia following bilateral thyroid surgery: A systematic review and meta-analysis. Thyroid Off. J. Am. Thyroid Assoc..

[7] Pelizzo M.R., Pagetta C., Piotto A., Sorgato N., Merante Boschin I., Toniato A., Grassetto G., Rubello D. (2008). Surgical treatment of primary hyperparathyroidism: From bilateral neck exploration to minimally invasive surgery. Minerva Endocrinol..

[8] Celik O., Yildiz B.O. (2021). Obesity and physical exercise. Minerva Endocrinol..

[9] Benaiges D., Goday A., Pedro-Botet J., Mas A., Chillaron J.J., Flores-Le Roux J.A. (2015). Bariatric surgery: To whom and when?. Minerva Endocrinol..

[10] Colquitt J.L., Pickett K., Loveman E., Frampton G.K. (2014). Surgery for weight loss in adults. Cochrane Database Syst. Rev..

[11] Diego Foschi M.D.L., Sarro G., Bernante P., Zappa M.A., Moroni R., Navarra G., Foletto M., Ceriani V., Piazza L., Di Lorenzo N. (2016). Linee Guida di Chirurgia Dell’obesità. https://www.sicob.org/00_materiali/linee_guida_2016.pdf.

[12] Chereau N., Vuillermet C., Tilly C., Buffet C., Tresallet C., du Montcel S.T., Menegaux F. (2017). Hypocalcemia after thyroidectomy in patients with a history of bariatric surgery. Surg. Obes. Relat. Dis. Off. J. Am. Soc. Bariatr. Surg..

[13] Dequanter D., Shahla M., Deniz Y., Aubert C., Lothaire P. (2016). Recalcitrant hypocalcemia after total thyroidectomy and bariatric surgery. B-ENT.

[14] (2013). National Code on Clinical Researches Published in Official Gazette (GU Serie Generale n.96 del 24-04-2013.). https://www.gazzettaufficiale.it/eli/id/2013/04/24/13A03474/sg.

[15] Pacini F., Basolo F., Bellantone R., Boni G., Cannizzaro M.A., De Palma M., Durante C., Elisei R., Fadda G., Frasoldati A. (2018). Italian consensus on diagnosis and treatment of differentiated thyroid cancer: Joint statements of six Italian societies. J. Endocrinol. Investig..

[16] Goldenberg D., Ferris R.L., Shindo M.L., Shaha A., Stack B., Tufano R.P. (2018). Thyroidectomy in patients who have undergone gastric bypass surgery. Head Neck.

[17] Spartalis E., Thanassa A., Athanasiadis D.I., Schizas D., Athanasiou A., Zografos G.N., Tsourouflis G., Dimitroulis D., Nikiteas N. (2019). Post-thyroidectomy Hypocalcemia in Patients with History of Bariatric Operations: Current Evidence and Management Options. In Vivo.

[18] Johnson J.M., Maher J.W., DeMaria E.J., Downs R.W., Wolfe L.G., Kellum J.M. (2006). The long-term effects of gastric bypass on vitamin D metabolism. Ann. Surg..

[19] Castagneto Gissey L., Casella Mariolo J.R., Genco A., Troisi A., Basso N., Casella G. (2018). 10-year follow-up after laparoscopic sleeve gastrectomy: Outcomes in a monocentric series. Surg. Obes. Relat. Dis. Off. J. Am. Soc. Bariatr. Surg..

[20] Barrea L., Colao A., Savastano S., Muscogiuri G. (2020). Specific cut-off for the 25-OH vitamin D levels to predict the highest Body Mass Index and fat mass: A sex-related analysis in obese patients. Minerva Endocrinol..

[21] Barrea L., Frias-Toral E., Pugliese G., Garcia-Velasquez E., de Los Angeles Carignano M., Savastano S., Colao A., Muscogiuri G. (2021). Vitamin D in obesity and obesity-related diseases: An overview. Minerva Endocrinol..

[22] Chakhtoura M., Rahme M., El-Hajj Fuleihan G. (2017). Vitamin D Metabolism in Bariatric Surgery. Endocrinol. Metab. Clin. N. Am..

[23] Nielsen M.S., Schmidt J.B., le Roux C.W., Sjodin A. (2019). Effects of Roux-en-Y Gastric Bypass and Sleeve Gastrectomy on Food Preferences and Potential Mechanisms Involved. Curr. Obes. Rep..

[24] Al-Najim W., Docherty N.G., le Roux C.W. (2018). Food Intake and Eating Behavior After Bariatric Surgery. Physiol. Rev..

[25] Mechanick J.I., Apovian C., Brethauer S., Timothy Garvey W., Joffe A.M., Kim J., Kushner R.F., Lindquist R., Pessah-Pollack R., Seger J. (2020). Clinical Practice Guidelines for the Perioperative Nutrition, Metabolic, and Nonsurgical Support of Patients Undergoing Bariatric Procedures—2019 Update: Cosponsored by American Association of Clinical Endocrinologists/American College of Endocrinology, The Obesity Society, American Society for Metabolic and Bariatric Surgery, Obesity Medicine Association, and American Society of Anesthesiologists. Obesity.

[26] Khan M.I., Waguespack S.G., Hu M.I. (2011). Medical management of postsurgical hypoparathyroidism. Endocr. Pract. Off. J. Am. Coll. Endocrinol. Am. Assoc. Clin. Endocrinol..

[27] Alfonso B., Jacobson A.S., Alon E.E., Via M.A. (2015). Previous gastric bypass surgery complicating total thyroidectomy. Ear Nose Throat J..

[28] McKenzie T.J., Chen Y., Hodin R.A., Shikora S.A., Hutter M.M., Gaz R.D., Moore F.D., Lubitz C.C. (2013). Recalcitrant hypocalcemia after thyroidectomy in patients with previous Roux-en-Y gastric bypass. Surgery.

[29] Durr M.L., Saunders J.R., Califano J.A., Tufano R.P., Koch W.M., Ha P.K. (2009). Severe hypocalcemia complicating thyroid surgery after Roux-en-Y gastric bypass procedure. Arch. Otolaryngol.-Head Neck Surg..

